# Understanding post-surgical decline in left ventricular function in primary mitral regurgitation using regression and machine learning models

**DOI:** 10.3389/fcvm.2023.1112797

**Published:** 2023-04-21

**Authors:** Jingyi Zheng, Yuexin Li, Nedret Billor, Mustafa I. Ahmed, Yu-Hua Dean Fang, Betty Pat, Thomas S. Denney, Louis J. Dell’Italia

**Affiliations:** ^1^ Department of Mathematics and Statistics, Auburn University, Auburn, AL, United States; ^2^ Division of Cardiovascular Disease, University of Alabama at Birmingham, Birmingham, AL, United States; ^3^ Department of Radiology, University of Alabama at Birmingham, Birmingham, AL, United States; ^4^ Birmingham Veterans Affairs Health Care System, Birmingham, AL, United States; ^5^ Department of Electrical and Computer Engineering, Samuel Ginn College of Engineering, Auburn University, Auburn, AL, United States

**Keywords:** machine learning, mitral regurgitation (MR), predictive models, LV circumferential strain rate, post-surgical LVEF

## Abstract

**Background:**

Class I echocardiographic guidelines in primary mitral regurgitation (PMR) risks left ventricular ejection fraction (LVEF) < 50% after mitral valve surgery even with pre-surgical LVEF > 60%. There are no models predicting LVEF < 50% after surgery in the complex interplay of increased preload and facilitated ejection in PMR using cardiac magnetic resonance (CMR).

**Objective:**

Use regression and machine learning models to identify a combination of CMR LV remodeling and function parameters that predict LVEF < 50% after mitral valve surgery.

**Methods:**

CMR with tissue tagging was performed in 51 pre-surgery PMR patients (median CMR LVEF 64%), 49 asymptomatic (median CMR LVEF 63%), and age-matched controls (median CMR LVEF 64%). To predict post-surgery LVEF < 50%, least absolute shrinkage and selection operator (LASSO), random forest (RF), extreme gradient boosting (XGBoost), and support vector machine (SVM) were developed and validated in pre-surgery PMR patients. Recursive feature elimination and LASSO reduced the number of features and model complexity. Data was split and tested 100 times and models were evaluated *via* stratified cross validation to avoid overfitting. The final RF model was tested in asymptomatic PMR patients to predict post-surgical LVEF < 50% if they had gone to mitral valve surgery.

**Results:**

Thirteen pre-surgery PMR had LVEF < 50% after mitral valve surgery. In addition to LVEF (*P* = 0.005) and LVESD (*P* = 0.13), LV sphericity index (*P* = 0.047) and LV mid systolic circumferential strain rate (*P* = 0.024) were predictors of post-surgery LVEF < 50%. Using these four parameters, logistic regression achieved 77.92% classification accuracy while RF improved the accuracy to 86.17%. This final RF model was applied to asymptomatic PMR and predicted 14 (28.57%) out of 49 would have post-surgery LVEF < 50% if they had mitral valve surgery.

**Conclusions:**

These preliminary findings call for a longitudinal study to determine whether LV sphericity index and circumferential strain rate, or other combination of parameters, accurately predict post-surgical LVEF in PMR.

## Introduction

Patients with primary mitral regurgitation (PMR) and left ventricular ejection fraction (LVEF) > 60% have a 20% chance of LVEF < 50% after mitral valve repair or replacement (1–3). Current Class I guidelines include conventional echocardiography-derived LVEF < 60%, LV end-systolic dimension > 4.0 cm or symptoms for surgical intervention (4). These guidelines were based on postoperative survival with less emphasis on postoperative LV function (1). We have demonstrated severe cardiomyocyte ultrastructural damage in patients with moderate to severe PMR and echocardiographic LVEF > 60% in PMR patients (5–7). These findings reinforce the concept that unrecognized cardiomyocyte ultrastructural damage may in part explain the decrease in post-operative LVEF.

Given the risk of waiting too long for surgery, current Class IIa indication for asymptomatic patients with severe PMR and LVEF > 60% recommends mitral valve repair at a Heart Valve Center of Excellence with a greater than 95% likelihood of a successful and durable repair without residual mitral regurgitation and expected mortality rate of less than 1% (4). However, outcomes among these asymptomatic patients are heterogeneous, and models to select the subset of asymptomatic patients with LVEF > 60% who will benefit from early surgery remains elusive. The impetus for the current study is to identify cardiac magnetic resonance (CMR) markers of LV function and remodeling to optimize timing of surgical intervention to reduce the likelihood of post-surgical decline in LVEF.

The advantages of machine learning models are their ability to integrate predictors extracted from multiple sources and to model both linear and nonlinear interactions amongst them (8). An important advantage of machine learning over conventional statistical methods (e.g., logistic regression) is that various machine learning algorithms do not require data to conform to statistical assumptions. Thus, machine learning models can identify unexpected predictors not accounted for by linear models and interactions that have prognostic value. With recursive feature elimination and repetitive testing, machine learning models are now being used in pilot analyses even in smaller data sets (9, 10).

**Figure 1:**
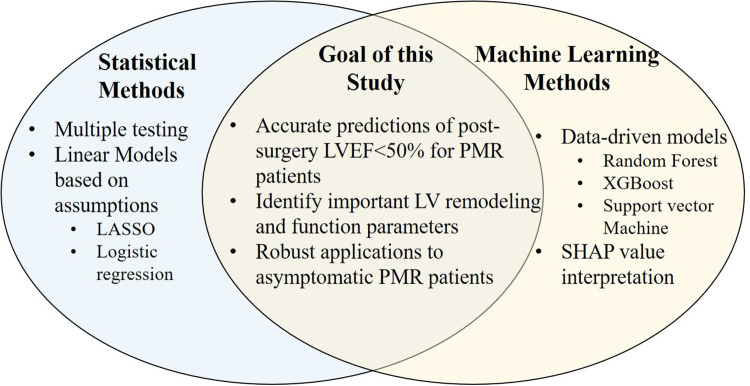
Application of regression and machine learning methods to achieve optimal analytical goals.

Recent studies employ machine learning and regression methods to achieve optimal analytical goals with multiple potential predictors (Figure 1) (11). The current study employs Random Forest (RF) (12), Support Vector Machine (SVM) with Radial Basis Function Kernel (13), extreme gradient boosting (XGBoost) (14), in addition to a standard least absolute shrinkage and selection operator (LASSO) penalized logistic regression (15). These models are trained, re-trained based on a reduced number of features and validated in data from pre-surgical PMR patients to predict six-month post-surgery LVEF < 50%. The final model is then applied to predict LVEF < 50% in a cohort of asymptomatic PMR patients if they had gone to mitral valve surgery.

## Materials and methods

### Study population

This single-center study includes 49 asymptomatic and 51 pre-surgery PMR patients recruited between 2006 and 2010 under NHLBI Specialized Centers of Clinically Oriented Research grant (5, 6, 16). Primary degenerative mitral valve prolapse characterized by echocardiographic evidence of thickened, redundant leaflets with excessive motion and prolapse. Patients were excluded for evidence of: (1) aortic valve > trace aortic regurgitation or mean gradient of >10 mmHg, (2) mitral stenosis (mean gradient > 5 mmHg, valve area < 1.5 cm^2^), (3) endocarditis, (4) iatrogenic MR (ergot, radiation induced), (5) hemodialysis, (6) pregnancy, (7) presence of coronary artery disease (stenosis > 50%), 8) positive exercise tolerance test with myocardial perfusion. The Institutional Review Boards of the University of Alabama at Birmingham and Auburn University approved the study protocol. All participants gave written informed consent.

### Data collection

All data from patients’ baseline and return visits were obtained prospectively and recorded in electronic health data records. Cardiac magnetic resonance (CMR) imaging with tissue tagging was performed in control volunteers who had no prior history of cardiovascular disease or medical illness, no history of smoking, and no cardiovascular medications. Asymptomatic PMR patients had Class I status, with moderate/severe PMR by color flow Echo/Doppler, LVEF > 60%, LV end-systolic dimensions (ESD) < 40 mm, leaflet thickening and prolapse, and normal maximal exercise myocardial perfusion imaging (17). At baseline, PMR patients (asymptomatic, *n* = 49 and pre-surgery, *n* = 51), plasma xanthine oxidase (XO) activity and carboxy-terminal propeptide of procollagen type I (PICP), a marker of type I collagen synthesis, and carboxy-terminal telopeptide of collagen type I (ICTP) levels, a marker of type I collagen degradation were measured. Post-surgical CMR was performed six months after the surgical procedure in all pre-surgery patients.

### Cardiac magnetic resonance

Table 1 lists all CMR-derived LV and left atrial volumes, strains and twist as previously described in our laboratory (5, 6, 16, 17).

**Table 1:** Features included in predictive models (*N* = 37).

Category (No. of Features)	Features
Demographics (9)	Age, Race, Gender, Weight, Height, BMI, BSA, Hypertension, Atrial Fibrillation
CMR	*Pre-surgery and Post-surgery (6 months)*
- LV function (6)	LV end-diastolic volume, LV end-systolic volume, LV end-diastolic dimension, LV end-systolic dimension, LVEF, LV Stroke Volume,
- LV remodeling (4)	LV end-diastolic mass, LV mass/volume, LV Sphericity Index (SI), LV mass/volume x SI
- Regurgitation (1)	Regurgitant Volume
- LA remodeling (3)	LA maximum and minimum volumes, Total LA emptying fraction
- RV parameters (1)	RV ejection fraction
LV CMR tissue tagging (9)	LV mid Systolic Circumferential Strain
	LV mid Systolic Longitudinal Strain
	LVES Maximum Strain
	LV mid Systolic Circumferential Strain Rate
	LV Systolic Longitudinal Strain Rate
	LV Peak Systolic Twist
	LV Systolic Twist-per-Volume Slope
	CL-Shear Angle
	LV Systolic Torsion
Biomarkers (4)	XOCM, PICP, ICTP, PICP/ICTP

CL, circumferential-longitudinal; CMR, cardiac magnetic resonance; ICTP, Carboxy-terminal telopeptide of collagen type I, a marker of type I collagen degradation; LA, left atrial; LV, left ventricle; RV, right ventricle; LVEF, LV ejection fraction; PICP, Carboxy-terminal propeptide of procollagen type I, a marker of type I collagen synthesis; XO, xanthine oxidase normalized to plasma protein (XOCM).

### Xanthine oxidase measurement

Peripheral venous XO activity was measured by the rate of uric acid production in the presence of xanthine (75 *μ*M) without NAD^+^ as described previously in our laboratory (16).

### Collagen homeostasis

Baseline levels of PICP and ICTP were measured with commercially available immunoassays (Quidel Corporation, USA and Orion Diagnostic, Finland). Detection limits were 0.2 ng/ml for PICP and 0.3 ng/ml for ICTP as described previously in our lab (16).

### Statistical analysis

Data in Tables are presented as number (% of total) or median (interquartile range). Statistical differences between two groups are tested *via* Mann-Whitney U test for continuous variables and chi-square test for categorical variables (Tables 1, 2 and Supplementary Table S1). Comparisons between 3 groups are tested by Kruskal-Wallis and the *p*-values adjusted by false discovery rate (FDR) for multiple testing, reported in Supplementary Table S2. Univariate and multivariate logistic regression are fitted to predict the probability of post-surgical LVEF < 50% using 4 pre-surgical parameters (LVEF, LVESD, LV sphericity index, and LV systolic circumferential strain rate (Supplementary Tables S3, S4).

**Table 2:** Pre-Surgery baseline demographic and CMR data separated by LVEF≥ or <50% at 6 months post-surgery (*N* = 51).

	LVEF ≥ 50% (*N* = 38)	LVEF < 50% (*N* = 13)	*P* value	FDR adjusted *P*-value
Age	56 (46, 62)	51 (43, 66)	0.85	0.91
Female/Male	8 (21%)/30 (79%)	7 (54%)/6 (46%)	**0.025**	0.204
BMI (kg/m^2^)	27 (24, 29)	25 (23, 30)	0.53	0.699
BSA (m^2^)	2.02 (1.89, 2.12)	1.80 (1.68, 2.04)	0.06	0.223
Hypertension (Y/N)	15 (39%)/23 (61%)	3 (23%)/10 (77%)	0.29	0.459
Atrial Fibrillation (Y/N)	5 (13%)/33 (87%)	5 (38%)/8 (62%)*	**0.047**	0.204
LVEF (%)	65 (62, 68)	58 (53, 64)*	**0.005**	0.146
LVED Volume (mL/m^2^)	104 (88, 128)	104 (96, 122)	0.91	0.91
LVES Volume (mL/m^2^)	36 (29, 45)	44 (36, 54)*	**0.036**	0.204
LV Stroke Volume (mL/m^2^)	69 (55, 82)	64 (54, 70)	0.23	0.459
LVED Diameter (mm)	57 (53, 62)	59 (55, 66)	0.30	0.459
LVES Diameter (mm)	44 (40, 47)	49 (40, 55)	0.13	0.376
LVED Mass/Volume (g/mL)	0.6 (0.6, 0.7)	0.6 (0.5, 0.7)	0.54	0.669
LV Sphericity Index (SI)	1.58 (1.43 1.78)	1.47 (1.29, 1.65)*	**0.047**	0.204
LVED Mass/Volume x SI	1.0 (0.8, 1.3)	0.9 (0.8, 1.1)	0.17	0.425
LV Systolic Twist/Volume slope (°/ml)	−0.07 (−0.09, −0.05)	−0.07 (−0.10, −0.05)	0.81	0.91
LV Systolic Circumferential Strain rate (1/s)	−0.69 (−0.77, −0.64)	−0.59 (−0.74, −0.51)*	**0.024**	0.204
Peak LV Torsion (°/cm)	2.05 (1.62, 2.55)	1.97 (1.34, 2.18)	0.30	0.459
Circumferential L-Shear Angle (°)	7.4 (6.5, 9.4)	7.1 (5.3, 8.8)	0.27	0.459
LVES Circumferential Strain	−0.15 (−0.16, −0.13)	−0.14 (−0.16, −0.12)	0.23	0.459
LVES Longitudinal Strain	−0.13 (−0.14, −0.11)	−0.11 (−0.16, −0.10)	0.89	0.91
LVES Maximal Strain	−0.20 (−0.21, −0.19)	−0.19 (−0.21, −0.18)	0.42	0.575
LA Max Volume (mL/m^2^)	58 (49, 81)	58 (53, 79)	0.76	0.898
LA Min Volume (mL/m^2^)	37 (22, 47)	38 (31, 47)	0.41	0.575
LA Emptying Fraction (%)	46 (37, 51)	42 (38, 46)	0.18	0.425
Regurgitant Volume (mL)	68 (49, 85)	57 (36, 70)	0.07	0.228

LV, left ventricle; LVED, LV end-diastolic; LVES, LV end-systolic; LVEF, LV ejection fraction; LA, left atrial; XO, xanthine oxidase normalized to plasma protein (XOCM) or plasma volume (XOCV); PICP, Carboxy-terminal propeptide of procollagen type I, a marker of type I collagen synthesis; ICTP, Carboxy-terminal telopeptide of collagen type I, a marker of type I collagen degradation.

Bold values indicate significance of *p* < 0.05.

### Model development in pre-surgical patients

**Figure 2:**
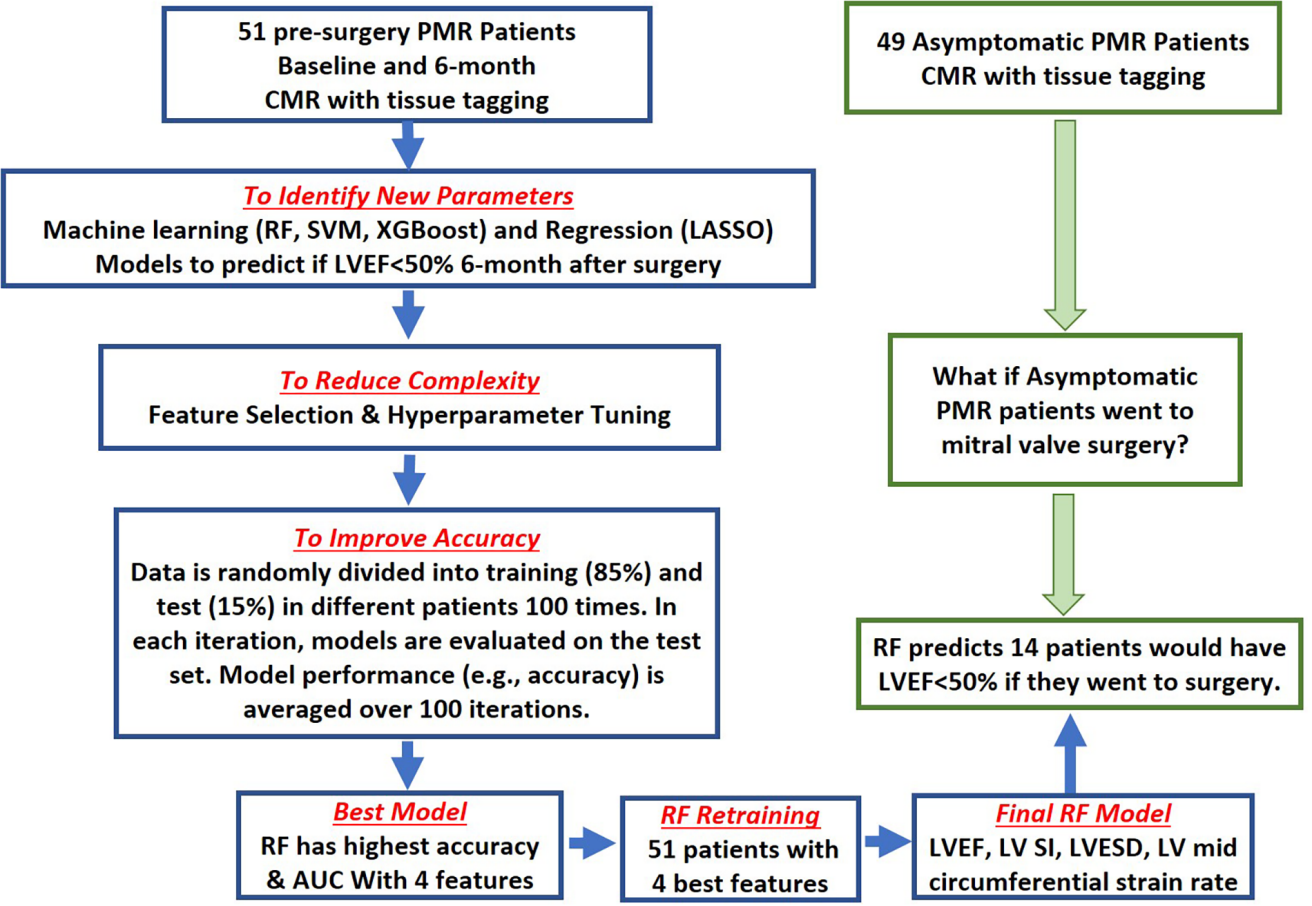
Flow chart of proposed model training and validation process. 51 pre-surgery and 49 asymptomatic PMR patients were recruited between 2005 and 2010. Random sampling was performed in pre-surgical patients to generate balanced data. Training (*n* = 43) and Testing Sets (*n* = 8) were established (blue flow chart). For each model, feature selection identified a subset of features relevant in predicting post-surgical LVEF <50% using LASSO, or the machine learning models: random forest (RF), support vector machine (SVM), and extreme gradient boosting (XGBoost) models. Random forest had the highest area under the curve and accuracy and was chosen as the best predictive model. Based on the top 4 predictive features [LVEF, LV Sphericity Index (SI), LVESD and LV systolic circumferential strain rate], the RF model was retrained on the 51 pre-surgery PMR patents then applied to 49 asymptomatic PMR patients to predict LVEF < 50% (14 patients—28%) if they were to have mitral valve surgery (green flow chart).

This study employed a standard LASSO (15) logistic regression and three machine learning models: random forest (RF) (12), support vector machine (SVM) with Radial Basis Function (RBF) Kernel (13), and extreme gradient boosting (XGBoost) (14). The models are trained to predict a binary response variable with two levels: LVEF < 50% or >50% at 6 months after mitral valve surgery in pre-surgery PMR patients. The models were initially developed using 37 features including demographics, CMR parameters, and biomarkers for predictive modeling (Table 1 and Figure 2).

### Feature selection

**Figure 3:**
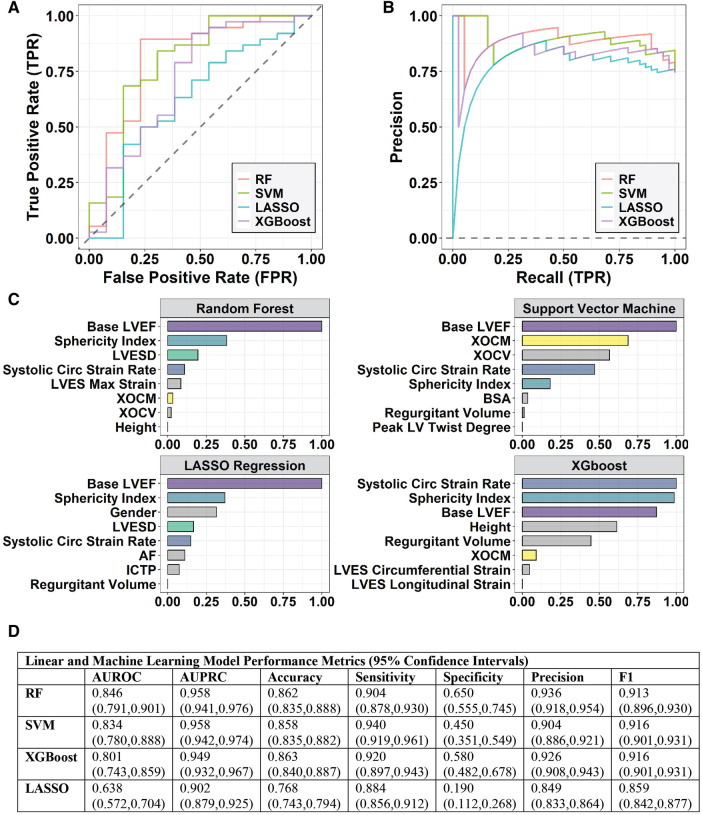
(**A**) the receiver operating characteristic (ROC) and (**B**) precision-recall curves (PRC) for logistic regression with LASSO, random forest (RF), SVM, and XGBoost models. (**C**) Feature selection identifies a subset of features that are relevant/important in predicting a post-surgical LVEF < 50% using LASSO for the linear model and Recursive Feature Elimination for the RF, SVM, and XGBoost models. XOCM is XO activity in *μ*Units/mg protein; XOCV is XO activity in μUnits/ml plasma. (**D**) Table of Model Performance for each Predictive Model with 95% confidence intervals in parentheses.

To avoid overfitting and to increase the reproducibility of the models, we attempted to include the most relevant features in predicting post-surgery LVEF < 50%. LASSO, which is a regression model penalized on $l_{1}$ penalty, performs both feature selection and regularization by itself to enhance the interpretability and accuracy of the linear model. Therefore, LASSO does not require extra feature selection. However, for RF, SVM, and XGBoost models, the most relevant features are selected by recursive feature elimination algorithm (18, 19). This fits machine learning models with all features at the beginning and excludes the least important features based on their importance rank, resulting in a model with a reduced number of features (Figure 2). This elimination process is then iterated by updating the feature importance rank using the model from the previous iteration, thus updating the model with a fewer number of features. The recursive feature elimination stops when the highest area under the ROC curve (AUROC) is achieved, and the final feature sets is the optimal feature set for our final models (Figure 3A).

For RF and XGBoost, the feature importance is measured by a mean decrease in Gini index, which reflects the contribution of each feature to the purity of the nodes or leaves in the tree-based models. The greater the mean decrease in Gini index, the more important the feature is in the model. For SVM, the feature importance is measured using the AUROC value. To compare the feature importance returned by different models, we scale the importance scores to the same scale (0–1) for each model separately as follows:

$$Imp_{i,scaled}=\frac{Imp_{i}-Imp_{min}}{Imp_{max}-Imp_{min}},i=1,2,\ldots ,\;37$$

where Imp is the raw importance score, $Imp_{max}$ is the maximum importance score among 37 features, and $Imp_{min}$ is the minimum importance score among 37 features. Figure 3C shows the top features selected by the four models.

### Model training and testing process

Fifteen percent of pre-surgical PMR patients are randomly selected as an independent testing set (*n* = 8), and the remaining 85% are used to train the initial machine learning models (*n* = 43) using the initial 37 features (Table 1). During each iteration in recursive feature elimination, hyper-parameter tuning and cross-validation are performed to obtain the model with a reduced feature set and optimal hyper-parameters. For the LASSO and machine learning methods, the corresponding models with the optimal feature sets that result in the highest AUROC were selected as final models. Subsequently, the four final models are further evaluated and compared *via* cross-validation. To eliminate the potential sampling bias and to obtain a robust model performance, we repeated the splitting, training, and testing process 100 times (i.e., using a different testing patient set of *n* = 8 vs. the training set of *n* = 43), and record the averaged model performance across iterations in the results.

The LASSO and machine learning models are evaluated *via* six metrics: classification accuracy, area under the receiver operating characteristic curve (AUROC), area under Precision-Recall (PR) curve (AUPRC), sensitivity, specificity, precision, and F1 score in the 51 pre-surgical PMR patients who have an actual known outcome of LVEF < 50% at 6 months post-surgery (Figure 3D). Among the six metrics, AUROC was used to choose the final prediction model for post-surgical LVEF < 50%.

$$Accuracy=\frac{TP+TN}{TP+TN+FP+FN}$$

$$Sensitivity=\frac{TP}{TP+FN}$$

$$Specificity=\frac{TN}{TN+FP}$$

$$Precision=\frac{TP}{TP+FP}$$

$$F1\;score=\frac{2TP}{2TP+FP+FN}$$

Where TP, TN, FP, and FN = true positive, true negative, false positive, and false negative.

### Interpretation of machine learning model

To interpret the final predictive model, we compute the Shapley Additive exPlanations (SHAP) (14) value, which is developed from the Shapley value in cooperative game theory. The Shapley value in game theory quantifies the contribution that each player brings to the game. Similarly, the SHAP value quantifies the contribution of each feature to the model prediction. In our case, one game is one patient, and the players are the optimal feature sets in the final predictive model. The SHAP value of a particular feature is computed by weighting the marginal contributions of the feature. For pre-surgery PMR patient or asymptomatic PMR patient, we compute the SHAP value to evaluate the contribution of each feature to prediction of the post-surgical LVEF < 50%. Therefore, the SHAP value provides a local (i.e., patient-level) interpretation of the decision made by the final machine learning model. Moreover, by averaging the absolute SHAP values, we can obtain the overall feature importance, which provides an overall (i.e., group-level) interpretation of the machine learning model. In addition, the sign of the SHAP value implies the directional impact of each feature on the prediction (i.e., a positive SHAP implies positive impact on the probability of post-surgical LVEF < 50%).

## Results

### Baseline characteristics of asymptomatic and pre-surgical PMR patients

There is a significantly higher incidence of episodic atrial fibrillation and medications, LV end-diastolic dimension (LVEDD), LVESD, and pulmonary artery systolic pressure (by Echo/Doppler) in pre-surgery vs. asymptomatic PMR. Forty seven per cent of pre-surgical patients are Class I, and all patients had normal renal function. Median pulmonary systolic pressure and median pulmonary artery wedge pressure are 38 and 16 mmHg, respectively, in pre-surgical patients (Supplementary Table S1).

### CMR in asymptomatic and pre-surgery patients with moderate to severe PMR

LVEF does not differ among normal and both PMR groups. LV end-diastolic volume, LV stroke volume, LVEDD, LVESD, and regurgitant volume and XO activity are greater in pre-surgery vs. asymptomatic PMR. However, LVED mass/volume, Sphericity Index, and LVED 3-dimensional radius of curvature/wall thickness at mid LV do not differ in asymptomatic and pre-surgery PMR patients. Asymptomatic and pre-surgery patients have decreased LV systolic twist/volume slope (°/mL) vs. controls. The increase in plasma ICTP and decrease in the PICP are consistent with net collagen degradation in pre-surgery PMR patients. There is an increase in LA maximum and minimum volumes in asymptomatic PMR compared to normal and they are higher in pre-surgical PMR patients. However, only pre-surgical PMR patients have a decrease in total LA emptying fraction (Supplementary Table S2).

### CMR in pre-surgery PMR patients with LVEF < 50% at 6 months post-surgery

Among the 51 pre-surgical patients, 13 patients (25.49%) had a decrease in CMR LVEF < 50% at 6 months post-surgery. Patients with LVEF < 50% were more likely to have a median baseline LVEF < 58% by CMR and a greater incidence of atrial fibrillation. Those with a decrease in LVEF had a higher LV end-systolic volume index and lower LV sphericity index and LV mid systolic circumferential strain rate, prior to surgery. However, FDR-adjusted *p*-values indicated no significant differences between the two groups (Table 2).

### Statistical and machine learning modeling in pre-surgery PMR patients

Four predictive models: a standard linear model - LASSO, and three machine learning models: RF, SVM, and XGBoost were trained to predict a binary response variable with two levels: LVEF < 50% or >50% at 6 months after mitral valve surgery in the 51 pre-surgery PMR patients (Figure 2). The models were initially developed using 37 features including demographics, CMR parameters, and biomarkers for predictive modeling (Table 1). To reduce the complexity of the model, feature selection (Figure 2) identifies a subset of features that are relevant in predicting a post-surgical LVEF < 50% using LASSO for the linear model and recursive feature elimination for the machine learning models. Figure 3A shows the ROC and precision-recall curves (PRC) (Figure 3B) and the top important features selected by LASSO and the machine learning models (Figure 3C) and lists the model performance (Figure 3D) for all four models including: AUROC, AUPRC, accuracy, sensitivity, specificity, precision, and F1 score along with the 95% confidence interval generated *via* bootstrapping. Overall, the machine learning models outperform the LASSO regression model. Based on the top features, the RF model provided the highest AUROC and AUPRC and was chosen to re-train the 51 pre-surgical patients using the four most relevant features identified by RF, for predicting LVEF < 50%: baseline LVEF, LV Sphericity Index, LVESD, and LV Systolic circumferential strain rate to improve predictability of the final model (Figure 2).

### Random forest model interpretation

**Figure 4:**
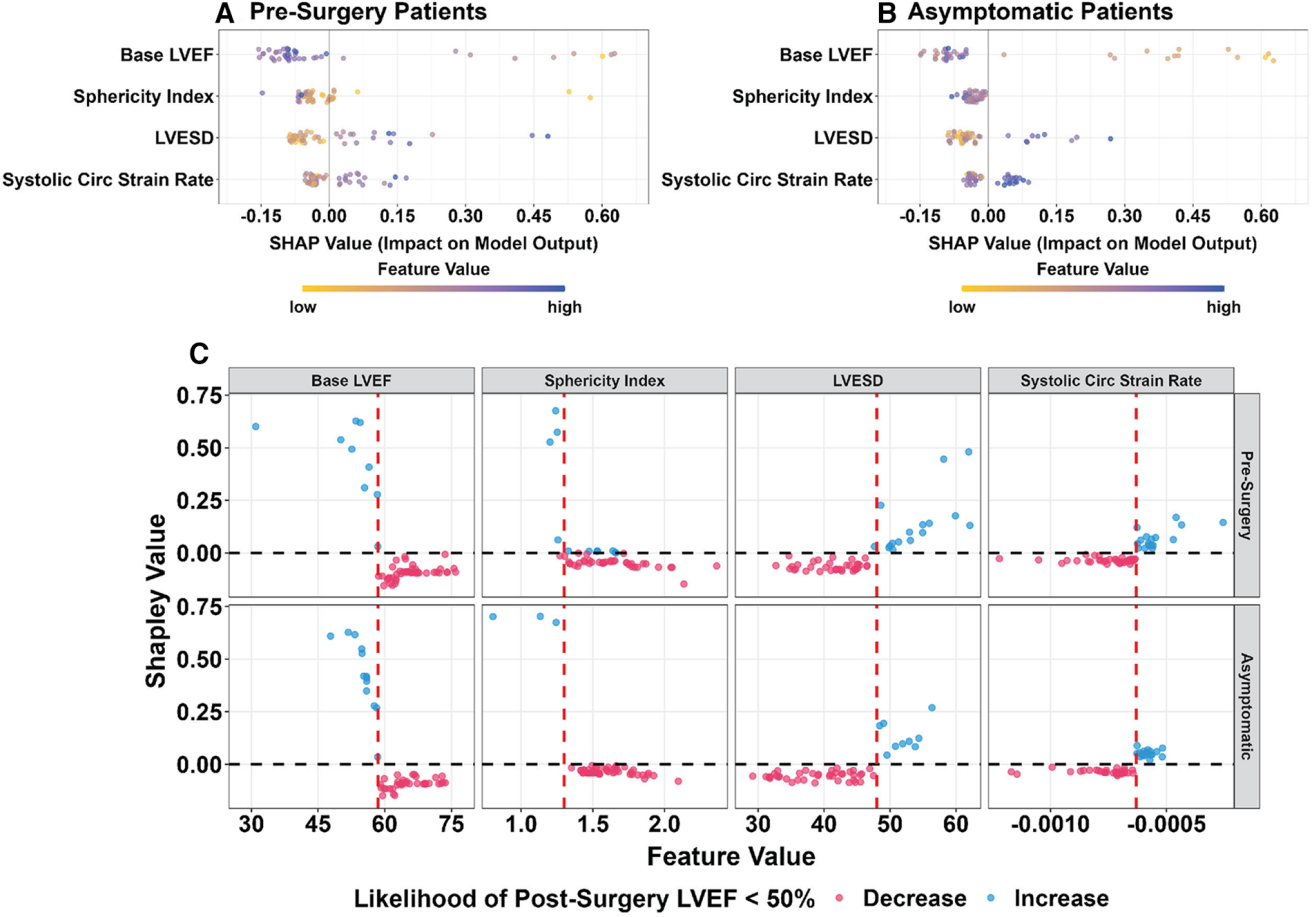
SHAP values for pre-surgery (**A**) and asymptomatic (**B**) PMR patients. Color value of each feature: highest blue and lowest yellow. Negative SHAP value (left side of 0.0) is a negative impact on the odds ratio, i.e., less likely to have post-surgical LVEF < 50%. Positive SHAP value (right side of 0.0) indicates a higher probability of post-surgical LVEF < 50%. (**C**) SHAP values for pre-surgery (top) & asymptomatic (bottom) PMR presented with potential cutoff values (red vertical dashed line) indicating the likelihood of post-surgical LVEF < 50%. A negative SHAP value (red) indicates a negative impact on the odds ratio, i.e., less likely to have post-surgical LVEF < 50%. A positive SHAP value (blue) indicates a higher probability of post-surgical LVEF < 50%. Each circle is representative of an individual patient.

The SHAP value interprets the contribution of the four important features of the RF model that predict post-surgery LVEF < 50% for both pre-surgery (Figure 4A) and asymptomatic PMR patients (Figure 4B). The directional impact of each feature is represented by the sign (negative or positive) of the SHAP value. A positive SHAP for each feature has a positive impact on the probability of post-surgery LVEF < 50%. A negative SHAP value has a negative impact on the probability of post-surgery LVEF < 50%. The larger the absolute value of SHAP, the greater the contribution the feature has to the model prediction.

Figure 4C shows the SHAP value of the four features in the RF model with a data-driven cutoff value (red vertical dashed line) indicating the directional impact on the likelihood of post-surgical LVEF < 50% in pre-surgery (top graphs) and asymptomatic (bottom graphs) PMR patients. Each circle represents one patient, and the color denotes positive (blue) or negative (red) impact of the feature on the probability (or likelihood) of post-surgery LVEF < 50%. The higher the absolute baseline CMR derived LVEF (>58%) and sphericity index (>1.3), the less likely for post-surgical LVEF < 50% (negative SHAP values). Mid LV systolic circumferential strain rate is a negative quantity and more negative values represent a greater circumferential shortening rate. Thus, <−0.63 1/s (−0.00063 1/ms) suggests that it is less likely for post-surgical LVEF < 50% (negative SHAP values). In comparison, the higher the LVESD (>48 mm), the more likely to have a post-surgical LVEF < 50% (positive SHAP value).

### Prediction of LVEF < 50% after mitral valve surgery in asymptomatic PMR

We address the important question of how many asymptomatic PMR patients would potentially be at risk for LVEF < 50% if they had gone to surgery. Therefore, we retrained the optimal RF model with the four selected features in all 51 pre-surgery PMR patients, and applied it to asymptomatic PMR patients (Figure 2). Random Forest predicted 14 out of 49 (28.57%) asymptomatic PMR patients would have LVEF < 50% post mitral valve surgery, had they gone to surgery.

## Discussion

In the current study, we employed a combination of machine learning and regression methods with the intention of identifying a combination of CMR (and biomarker) predictors not accounted for by a linear model alone for predicting LVEF < 50% at 6 months after surgery. In addition to LVEF and LVESD, mid LV systolic circumferential strain rate and LV sphericity index predict LVEF < 50% six months after surgery. Figure 1 outlines the value of utilizing both machine learning and regression modeling to achieve an optimal analytical result in addressing this complicated question. Compared with classic regression models, machine learning: (1) integrates predictors extracted from multiple sources and models both linear and nonlinear interactions amongst them and; (2) identifies unexpected predictors not accounted for by linear models.

The RF model was most accurate when reducing the features to LVEF, LVESD, LV sphericity index and LV mid wall circumferential strain rate in comparison to linear regression modeling. CMR LV strain emanates from tissue tagging that allows for intramyocardial displacement and strain by motion of identifiable material points distributed throughout the myocardium (5, 6). The adverse spherical remodeling, increase in LV mid radius/wall thickness, and global decrease in LVED mass/volume elevates wall stress resulting in a detrimental effect on LV mid circumferential shortening. Models that determine the effect of LV shape on LVEF demonstrate the importance of circumferential strain over longitudinal strain in maintaining LVEF in the spherically dilated LV (20). However, these models do not account for the presence of mitral regurgitation. In this pilot study, the RF model captures the inescapable LV spherical remodeling characteristic of PMR (5, 6, 16, 21). This connection to circumferential strain rate rather than circumferential strain alone further underscores the confounding factors of PMR ejection dynamics in the face of ejection into the low pressure left atrium, increased preload, and increased adrenergic drive in patients with PMR and LVEF > 60% (22, 23). The decrease in contractile velocity at the LV mid wall can be attributed to the loss of sarcomeres in the PMR heart (5, 6, 16, 21).

Regurgitant volume and plasma XO activity are other features identified more than once in the four models. However, the regurgitant volume calculated from the difference of LV and RV stroke volumes is an underestimate due to the 30% incidence of significant tricuspid regurgitation in the pre-surgical PMR patients. When calculated using phase velocity mapping for forward stroke volume, regurgitant volume may become a very powerful feature predictor as demonstrated in previous studies in PMR patients (24). We have reported an increase in LV and plasma XO, extensive mitochondrial damage, and breakdown of desmin in patients with moderate to severe PMR and LVEF > 60% (5, 6, 16). Xanthine oxidase can depress myofilament sensitivity to calcium and XO products like superoxide and hydrogen peroxide, can oxidatively influence mitochondria, myofilaments, calcium handling proteins, resulting in decreased LV strain rate (25).

An important addition to the interpretation of machine learning models is the SHAP (Shapley Additive exPlanations) (14) value, developed from the Shapley value in cooperative game theory. The SHAP value assigns each feature an importance value for a particular prediction to explain the decision made by the machine learning models. The SHAP value provides an *overall interpretation* of the machine learning models including a directional impact of each feature on the prediction (i.e., a positive or negative impact on the probability of post-surgical LVEF < 50%) and a *local interpretation* at the patient level (i.e., knowing how each feature contributes to an individual prediction for each patient). This provides cutoff values that in a larger sample size can comprise a risk score.

The relatively small sample size and the absence of an external validation set is a limitation in this preliminary study, in addition to the potential for overfitting in a small number of patients. To address this, feature selection *via* LASSO (linear model) or recursive feature elimination (machine learning models) reduces the number of features and model complexity. To eliminate the potential sampling bias and obtain a robust model performance, we iterate the splitting, training, and testing process 100 times (i.e., using a different testing patient set of *n* = 8 vs. the training set of *n* = 43), and record the averaged model performance.

To demonstrate the limitation of regression analysis, we employed univariate regression vs. multivariate regression for LVEF, LVESD, LV sphericity index and LV mid circumferential strain rate to predict LVEF < 50%. Coefficients for univariate regression with single features (Supplementary Table S3) produce a similar pattern to machine learning, and the four features except LVESD. Inclusion of the four features in a multivariate logistic regression, the coefficients still produce similar patterns as machine learning, however, all four features are no longer significant (Supplementary Table S4). Supplementary Table S5 also shows the odds ratio of the top 4 and 8 features from the RF model (Figure 3C). Therefore, multivariate logistic regression is not adequate when multiple features are interacting in both linear and nonlinear relations with the outcome. Consequently, the use of machine learning models may provide a better method of accurately assessing the contribution of linear and non-linear features in predicting a post-surgical decline in LVEF < 50%. This is depicted in a representative tree in the random forest (Supplementary Figure S1) and variable dependence and partial dependence plots (Supplementary Figure S2) that show that the association of the top 4 features in the RF model are not linear to the outcome (LVEF < 50%). This underscores the analytical benefits of a combination of regression and machine learning models and the potential advantages of utilizing the latter (Figure 1).

The impetus for this study is the unreliability of an echocardiographic LVEF > 60% in the complicated context of increased preload and facilitation of LV ejection in patients with PMR. Machine learning and multiple testing most certainly increase the risk of drawing a false-positive conclusion. Nevertheless, the findings from this approach, in albeit a small number of patients present a cogent argument for the underpinnings of LV sphericity and mid LV circumferential strain rate in the pathophysiology of PMR. Whether these, or any combination of LV and biomarker features, can reliably identify the need for surgery with better LV functional recovery can only be confirmed in a longitudinal study that incorporates machine learning and statistical models that capture both linear and non-linear interactions in asymptomatic PMR patients with LVEF > 60%.

## Data Availability

The original contributions presented in the study are included in the article/**Supplementary Material**, further inquiries can be directed to the corresponding author.
