# Supplementary material for: Understanding post-surgical decline in left ventricular function in primary mitral regurgitation using regression and machine learning models
Source: Front Cardiovasc Med. 2023 Apr 21;10:1112797. doi: 10.3389/fcvm.2023.1112797 (PMC10160646; doi:10.3389/fcvm.2023.1112797)

**Supplemental Material**

**Supplemental Tables**

| **Supplemental Table 1. Demographics of PMR Patients and Transthoracic Echo/Doppler.** | | | |
| --- | --- | --- | --- |
|  | **Asymptomatic**  **N = 49**  **No. (%)** | **Symptomatic**  **N = 51**  **No. (%)** | **FDR Adjusted p-value** |
| **No Medications** | **34 (72%)** | **27 (33%) *** | **0.0008** |
| **Beta blocker** | **3 (6%)** | **25 (31%) *** | **0.0081** |
| **ACE inhibitor** | **3 (6%)** | **19 (23%) *** | **0.0383** |
| **AT_1_ Receptor Blocker** | **1 (2%)** | **10 (12%)** | **0.09** |
| **Antiarrhythmic** | **1 (2%)** | **6 (7%)** | **0.4725** |
| **Anticoagulant** | **0** | **5 (6%)** | **0.24** |
| **Calcium Entry Blocker** | **1 (2%)** | **6 (7%)** | **0.4725** |
| **Diuretic** | **5 (10%)** | **22 (27%)*** | **0.0832** |
| **Statins** | **4 (9%)** | **10 (12%)** | **0.6035** |
| **Diabetes Mellitus** | **0** | **1 (2%)** |  |
| **Hypertension** | **5 (11%)** | **23 (28%)** | **0.0578** |
| **Atrial Fibrillation** | **3 (6%)** | **19 (23) *** | **0.0383** |
| **NYHA Class** |  |  | **<0.0001** |
| **Class I** | **45 (92%)** | **24 (47%)** |  |
| **Class II** | **2 (4%)** | **24 (47%)** |  |
| **Class III** | **0** | **<2 (4%)** |  |
| **Missing** | **2 (4%)** | **1 (2%)** |  |
| **Transthoracic Echo/Doppler** | | | |
| **LV end-diastolic dimension (mm)** | **55 (37, 52)** | **54 (51, 57) *** | **0.0284** |
| **LV end-systolic dimension (mm)** | **30 (28, 34)** | **36 (32, 40) *** | **0.0016** |
| **LVEF (%)** | **55 (55, 55)** | **55 (55, 55)** | **0.26** |
| **LA dimension (mm)** | **41 (39, 45)** | **44 (39, 47)** | **0.13** |
| **PA systolic pressure Doppler (mmHg)** | **33 (29, 38)** | **38 (31, 48) *** | **0.007** |
| **PA systolic pressure Indwell. Cath (mmHg)** |  | **33 (24, 47)** |  |
| **PA Wedge Pressure (mmHg)** |  | **16 (10, 20)** |  |
| **TR ≥ 2** | **4** | **15** | **0.66** |

ACE – angiotensin converting enzyme; AT_1_ – angiotensin II type 1; LA – left atrial; LV – left ventricle; LVEF – LV ejection fraction; PA – pulmonary artery; PMR – primary mitral regurgitation; TR – tricuspid regurgitation;

| **Supplemental Table 2. PMR Demographics and CMR Data.** **P-values *vs. Normal; # vs. Asymptomatic** | | | | | |
| --- | --- | --- | --- | --- | --- |
|  | **Normal**  **(57)** | **PMR**  **Asymptomatic**  **(49)** | **Pre-Surgery PMR Symptomatic**  **(51)** | **p-value** | **FDR adjusted**  **p-value** |
| **Age** | **48 (33, 56)** | **53 (45, 62) *** | **55 (45, 62) *** | **0.0008** | **0.0012** |
| **Female/Male** | **30(53%)/27(47%)** | **31(63%)/18(37%)** | **15(29%)/36(71%) *** | **0.0023** | **0.003** |
| **BMI (kg/m^2^)** | **24 (21, 28)** | **26 (22, 28)** | **27 (23, 30)** | **0.10** | **0.117** |
| **BSA (m^2^)** | **1.87 (1.70, 2.09)** | **1.83 (1.68, 2.05)** | **2.01 (1.80, 2.11)** | **0.11** | **0.119** |
| **LVEF (%)** | **64 (61, 67)** | **63 (58, 67)** | **64 (61, 67)** | **0.56** | **0.56** |
| **LVED Volume (mL/m^2^)** | **71 (61, 76)** | **91 (75, 103) *** | **104 (92, 124) *^#^** | **<0.0001** | **<0.0002** |
| **LVES Volume (mL/m^2^)** | **24 (21, 29)** | **33 (26, 40) *** | **37 (30, 48) *** | **<0.0001** | **<0.0002** |
| **LV Stroke Volume (mL/m^2^)** | **43 (37, 50)** | **57 (47, 64) *** | **66 (55, 79) *^#^** | **<0.0001** | **<0.0002** |
| **LVED Diameter (mm)** | **51 (48, 53)** | **54 (50, 59) *** | **57 (53, 64) *^#^** | **<0.0001** | **<0.0002** |
| **LVES Diameter (mm)** | **37 (34, 41)** | **42 (36, 46)** | **45 (40, 50) *^#^** | **<0.0001** | **<0.0002** |
| **LVED Mass/Volume** | **0.7 (0.6, 0.8)** | **0.6 (0.5, 0.7) *** | **0.6 (0.6, 0.7) *** | **0.0005** | **0.0008** |
| **LV Sphericity Index (SI)** | **1.72 (1.63, 1.86)** | **1.55 (1.46, 1.70) *** | **1.57 (1.40, 1.74) *** | **<0.0001** | **<0.0002** |
| **LVED Mass/Volume x SI** | **1.30 (1.05, 1.50)** | **1.00 (0.80, 1.10) *** | **1.00 (0.79, 1.18) *** | **<0.0001** | **<0.0002** |
| **LVED radius/wall thickness** | **3.76 (3.44, 4.18)** | **4.40 (4.05, 5.15) *** | **4.48 (3.85, 4.92) *** | **<0.0001** | **<0.0002** |
| **LA Max Volume (mL/m^2^)** | **31 (26, 38)** | **46 (34, 59) *** | **58 (50, 80) *^#^** | **<0.0001** | **<0.0002** |
| **LA Min Volume (mL/m^2^)** | **14 (11, 17)** | **21 (15, 33) *** | **37 (25, 47) *^#^** | **<0.0001** | **<0.0002** |
| **LA Emptying Fraction (%)** | **54 (48, 60)** | **51 (42, 58)** | **44 (38, 50) *^#^** | **<0.0001** | **<0.0002** |
| **Regurgitant Volume (mL)** | **―** | **39 (26, 53)** | **64 (45, 84) ^#^** | **<0.0001** | **<0.0002** |
| **Regurgitant Fraction (%)** | **―** | **39 (28, 50)** | **46 (38, 57) ^#^** | **0.01** | **0.0127** |
| **LV Systolic Twist/Vol slope (°/ml)** | **-0.11 (-0.16, -0.09)** | **-0.08 (-0.10, -0.06) *** | **-0.07 (-0.09, -0.05) *** | **<0.0001** | **<0.0002** |
| **LV Systolic Circum Strain rate (1/s)** | **-0.72 (-0.79, -0.65)** | **-0.69 (-0.77, -0.60)** | **-0.66 (-0.77, -0.59)** | **0.11** | **0.1185** |
| **LVES Circumferential Strain** | **-0.16 (-0.17, -0.14)** | **-0.14 (-0.15, -0.13) *** | **-0.14 (-0.16, -0.13) *** | **0.00036** | **0.0007** |
| **LVES Longitudinal Strain** | **-0.12 (-0.14, -0.11)** | **-0.14 (-0.15, -0.13) *** | **-0.13 (-0.15, -0.11)** | **0.00534** | **0.0071** |
| **LVES Maximal Strain** | **-0.19 (-0.21, -0.18)** | **-0.20 (-0.21, -0.19)** | **-0.20 (-0.21. -0.19)** | **0.49** | **0.51** |
| **XOCM activity (μU/mg)** | **0.006 (0, 0.021)** | **0.011 (0.004, 0.017)** | **0.018 (0.012, 0.035) *^#^** | **<0.0001** | **<0.0002** |
| **XOCV activity (μU/mL)** | **0.48 (0, 1.51)** | **0.75 (0.31, 1.37)** | **1.34 (0.71, 2.04) *^#^** | **0.0005** | **0.0008** |
| **PICP (Synthesis)** | **92 (64, 114)** | **89 (76, 108)** | **75 (64, 93) *** | **0.034** | **0.0414** |
| **ICTP (Degradation)** | **3.0 (2.0, 3.8)** | **2.7 (2.2, 3.4)** | **3.5 (2.8, 4.8) *^#^** | **0.0021** | **0.0031** |

LV – left ventricle; LVED – LV end-diastolic; LVES – LV end-systolic; LVEF – LV ejection fraction; LA – left atrial; XO – xanthine oxidase normalized to plasma protein (XOCM) or plasma volume (XOCV); PICP - Carboxy-terminal propeptide of procollagen type I, a marker of type I collagen synthesis; ICTP - Carboxy-terminal telopeptide of collagen type I, a marker of type I collagen degradation

| **Supplemental Table 3. Univariate logistic regression to predict post-LVEF < 50%** | | | |
| --- | --- | --- | --- |
| **Univariate** Logistic regression model | Feature | Coefficient | p-value |
| Model 1 | Base LVEF | **-0.189** | **0.009** |
| Model 2 | Sphericity Index | **-3.465** | **0.0485** |
| Model 3 | LVESD | **0.08057** | **0.0795** |
| Model 4 | LV Systolic Circum. Strain Rate | **6711.322** | **0.0275** |

| **Supplemental Table 4. Multivariate Logistic regression to predict post-LVEF<50%** | | |
| --- | --- | --- |
| **Feature included in the multivariate model** | **Coefficient** | **P-value** |
| Base LVEF | **-0.157**  **(-0.182, -0.132)** | **0.0806** |
| Sphericity Index | **-4.956**  **(-5.71, -4.201)** | **0.0647** |
| LVESD | **0.049**  **(0.033, 0.066)** | **0.4106** |
| LV Systolic Circum. Strain Rate | **6492.279**  **(5365.325, 7619.232)** | **0.1052** |

| Supplemental Table 5. Logistic regression with odds ratio for the top 4 and 8 features in the Random Forest Model. | | | | | | |
| --- | --- | --- | --- | --- | --- | --- |
|  | Logistic Regression with Top 4 Features from RF | | | Logistic Regression with Top 8 Features from RF | | |
| Feature | **Coefficient Estimate** | **Odds Ratio** | **P-value** | **Coefficient Estimate** | **Odds Ratio** | **P-value** |
| Base LVEF | -0.157 (-0.182, -0.132) | 0.855 | **0.0806** | -0.164 (-0.193, -0.136) | 0.848 | **0.1** |
| Sphericity Index | -4.956 (-5.71, -4.201) | 0.007 | **0.0647** | -5.472 (-6.236, -4.708) | 0.004 | **0.044** |
| LVESD | 0.049 (0.033, 0.066) | 1.051 | 0.4106 | 0.058 (0.041, 0.076) | 1.06 | 0.348 |
| Systolic Circ Strain Rate | 6492.279  (5365.325, 7619.232) | $e^{6492.279}$ | 0.1052 | 6692.217  (5424.482, 7959.952) | $e^{6692.217}$ | 0.138 |
| LVES Max Strain |  |  |  | -21.796 (-29.764, -13.829) | $3.419\times{10}^{-10}$ | 0.442 |
| XOCM |  |  |  | -205.974 (-250.52, 161.427) | $3.521\times{10}^{-90}$ | 0.193 |
| XOCV |  |  |  | 2.673 (2.091, 3.254) | 14.478 | 0.196 |
| Height |  |  |  | -0.014 (-0.025, -0.002) | 0.986 | 0.743 |

**Supplemental Figure 1**

Representative tree in the Random Forest. The leaves show the LVEF prediction along with the ground truth in Pre-Surgical Patients.


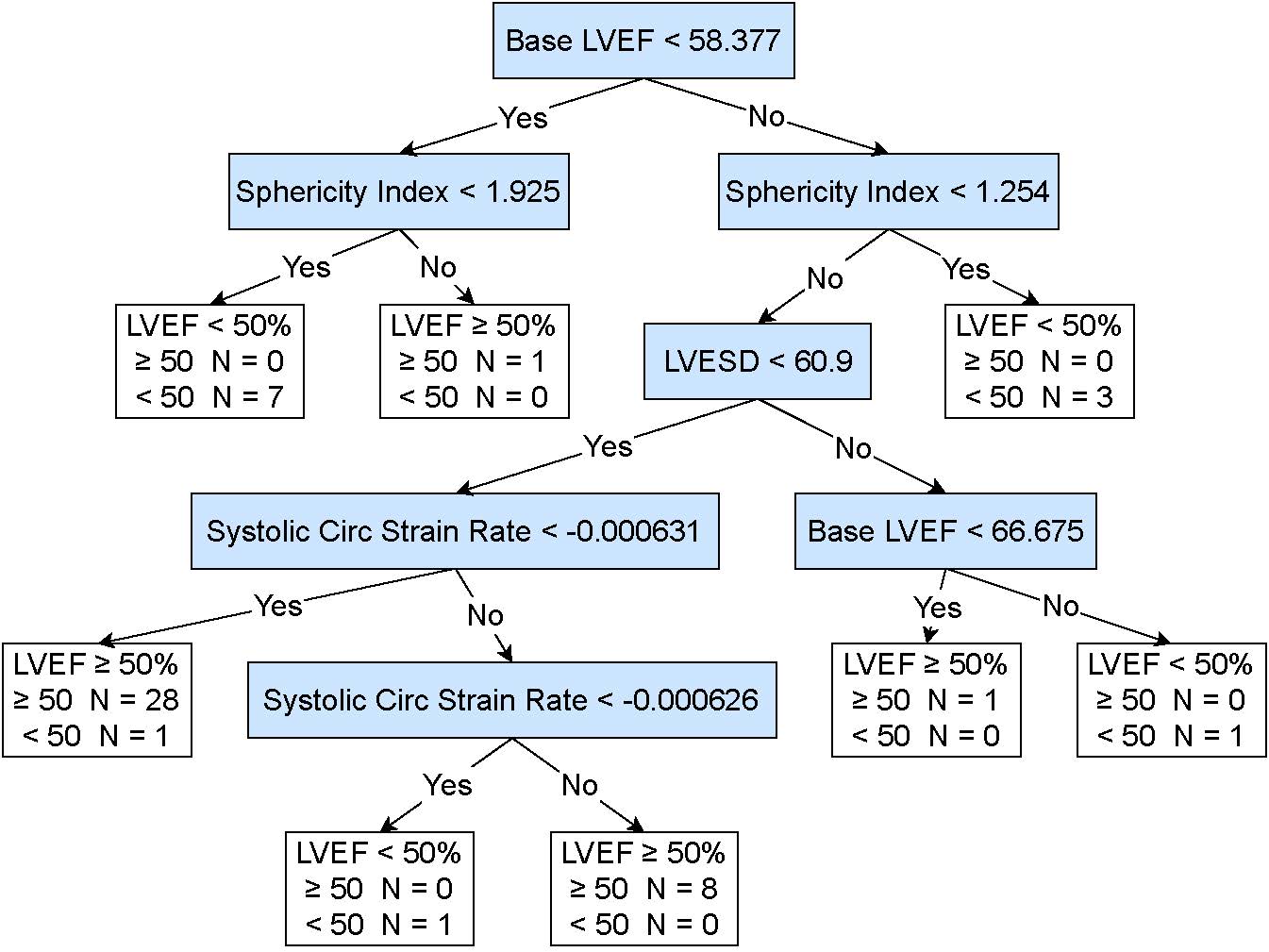


**Supplemental Figure 2**

Variable dependence (**A**) and partial dependence plots (**B**) depicting the non-linear relationships between outcome (LVEF > or < 50%) and the top 4 features in the Random Forest model – Baseline LVEF, LV sphericity index, LV end-systolic dimension and LV systolic circumferential strain rate. The colored dots represents the true label for each patient, which is the known outcome of post-surgical LVEF < 50% (yellow) or LVEF > 50% (green). The X-axis represents the value of each feature while the Y-axis is the predicted probability of post-surgical LVEF < 50%.

**A**


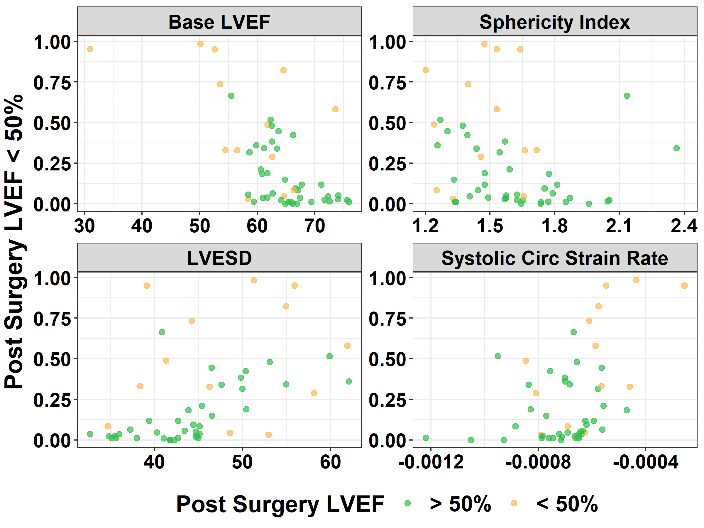

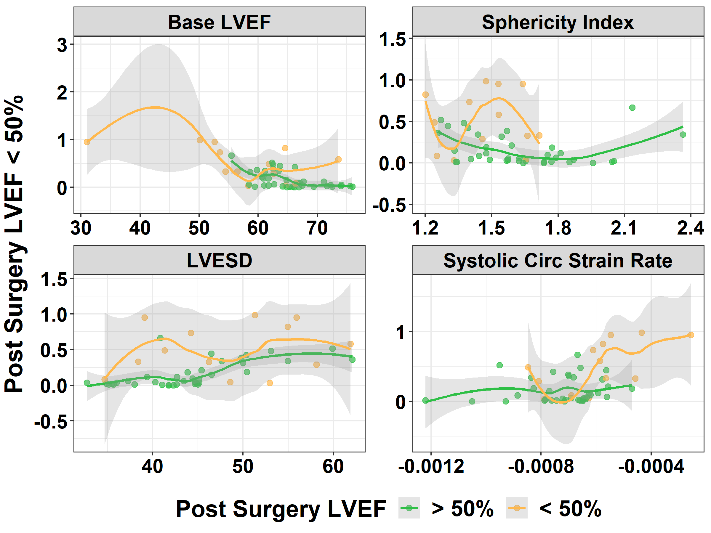


**B**


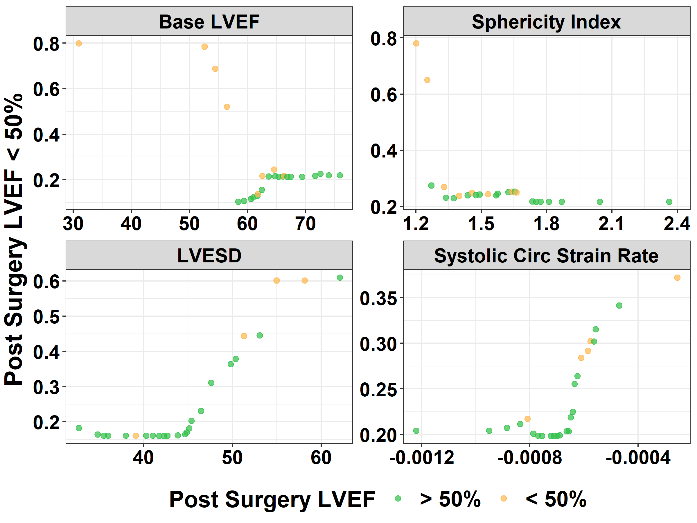

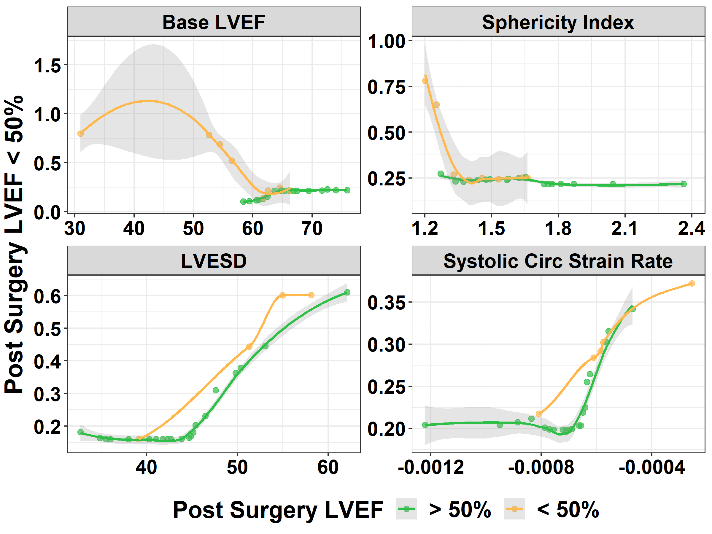

Supplement: Supplementary file 1 [file Datasheet1.docx]
